# Burden of female breast cancer in the Middle East and North Africa region, 1990–2019

**DOI:** 10.1186/s13690-022-00918-y

**Published:** 2022-07-11

**Authors:** Saeid Safiri, Maryam Noori, Seyed Aria Nejadghaderi, Mark J. M. Sullman, Nicola Luigi Bragazzi, Amir Almasi-Hashiani, Mohammad Ali Mansournia, Ali-Asghar Kolahi

**Affiliations:** 1 Research Center for Integrative Medicine in Aging, Aging Research Institute, Tabriz University of Medical Sciences, Tabriz, Iran; 2 Department of Community Medicine, Faculty of Medicine, Tabriz University of Medical Sciences, Tabriz, Iran; 3 Student Research Committee, School of Medicine, Iran University of Medical Sciences, Tehran, Iran; 4 Urology Research Center, Tehran University of Medical Science, Tehran, Iran; 5 School of Medicine, Shahid Beheshti University of Medical Sciences, Tehran, Iran; 6 Systematic Review and Meta-analysis Expert Group (SRMEG), Universal Scientific Education and Research Network (USERN), Tehran, Iran; 7 Department of Life and Health Sciences, University of Nicosia, Nicosia, Cyprus; 8 Department of Social Sciences, University of Nicosia, Nicosia, Cyprus; 9 Centre for Disease Modelling, York University, Toronto, Ontario Canada; 10 Department of Epidemiology, School of Health, Arak University of Medical Sciences, Arak, Iran; 11 Department of Epidemiology and Biostatistics, School of Public Health, Tehran University of Medical Sciences, Tehran, Iran; 12 Social Determinants of Health Research Center, Shahid Beheshti University of Medical Sciences, Tehran, Iran

**Keywords:** Breast Cancer, Neoplasms, Epidemiology, Incidence, Mortality, Middle East and North Africa

## Abstract

**Background:**

There is no region-specific publication investigating the attributable burden of breast cancer, particularly among females. This article reported the burden of female breast cancer in the Middle East and North Africa (MENA) region, and its attributable risk factors between 1990 and 2019, by age, sex, and socio-demographic index (SDI).

**Methods:**

Publicly available data on the incidence, death and disability-adjusted life years (DALY) were retrieved from the Global Burden of Disease (GBD) 2019 study for the 21 countries and territories in MENA, between 1990 and 2019, along with 95% uncertainty intervals (UIs). The relationship between the burden of female breast cancer, in terms of DALYs, and the SDI were also assessed using Smoothing Spline models.

**Results:**

In 2019, the regional age-standardised incidence and death rates of female breast cancer were 37.5 and 15.2 per 100,000, which represent a 90.9 and 24.0% increase since 1990, respectively. In addition, in 2019 the regional age-standardised DALY rate was 472.7 per 100,000, which was 19.5% higher than in 1990. In 2019, the death rate increased steadily with advancing age, while the DALY rate increased steeply with age and reached its peak in the 70–74 age group. There was a positive association between SDI and the burden of breast cancer over the period 1990 to 2019. Moreover, in 2019 high fasting plasma glucose (6.9%) contributed to the largest proportion of attributable DALYs for female breast cancer in the MENA region.

**Conclusions:**

There was a significant increase in the incidence rate of female breast cancer in MENA over the past three decades, although the death and DALY rates were both largely unchanged. Preventive programs targeting the major risk factors should be implemented in the region.

**Supplementary Information:**

The online version contains supplementary material available at 10.1186/s13690-022-00918-y.

## Introduction

According to the Global Burden Disease (GBD) study 2019, the survival rate from cancers has substantially improved over the last three decades, although the incidence rate is on the rise [1]. Among the cancers that mainly affect females, breast cancer is the most common globally. It is important to note, that of the 20.6 million disability-adjusted life years (DALY) due to breast cancer, 20.3 million were among women [1]. In 2019, breast cancer had the highest mortality rate among females, with more than 688 thousand deaths, and accounted for 15.9% of all cancer-related deaths [2]. Moreover, the global number of female breast cancer cases increased by 128.3% between 1990 and 2019 [2]. There were also large increases in the number of incident cases (377.9%), deaths (203.7%) and DALYs (197.2%) attributable to breast cancer in the Middle East and North Africa (MENA) region over the same period [2]. Globally, due to rapid population growth and aging, the prevalence and composition of the main risk factors have changed, resulting in substantial variations in the burden of breast cancer by region and country [3].

Breast cancer is most often clinically classified according to the molecular alterations. In general, tumors expressing estrogen or progesterone receptors are considered hormone receptor-positive breast cancers, whereas tumors that do not express hormone receptors or human epidermal growth factor receptor 2 (HER2) are triple negative breast cancers. The treatment of breast cancer and their prognosis are highly dependent upon the histological characteristics of the tumor cells [4]. Early breast cancer, defined as locally restricted tumor cells, is considered curable in around 70–80% of patients. In contrast, whenever the tumor become metastatic, the disease would be considered incurable with current therapeutic options [5].

Previous research, using GBD 2019 data, showed that in 2019 the global age-standardised incidence rate of breast cancer was 24.2 (22.1, 26.2) per 100,000 population, with a rate of 0.7 (0.6–0.7) for males and 45.9 (41.9–49.8) for females [1, 2]. Furthermore, the majority of the incidences and deaths occurred in women aged between 50 and 70 years old, accounting for about 50% of the total cases [2]. Furthermore, an overall positive relationship was found between a country’s developmental status and their breast cancer mortality rate [2]. In addition to the findings of the GBD 2019 study, a number of other articles have reported the burden of breast cancer at the global, regional, and national level, using GBD 2017 data [6, 7]. Nevertheless, there is no region-specific publication investigating the attributable burden of breast cancer, particularly among females. Furthermore, the GBD capstone papers aim to provide an overview of the global situation, in order to stimulate more detailed research on the burden of different diseases and injuries at the regional and national levels. Additionally, the findings of this study might help policymakers make decisions about how to allocate public health resources. Nevertheless, any interventions must also take into consideration the diverse socio-cultural and economic situation within the MENA region. As a result, we aimed to report the burden of female breast cancer and its attributable risk factors in the 21 countries located in the MENA region, by age, sex, and socio-demographic index (SDI) between 1990 and 2019.

## Methods

### Overview

GBD 2019 project collected data from 204 countries and territories regarding 369 diseases and injuries and 87 risk factors. GBD 2019 data was used to report the burden of female breast cancer in the MENA region, and the individual countries within this region, from 1990 to 2019. The estimation process has improved over the period 1990–2019, through the addition of more data sources and methodological improvements, which have previously been described in detail [8, 9]. There were 30 cancer groups estimated in GBD 2019, including breast cancer. All estimates and rates were reported per 100,000 women along with 95% uncertainty intervals (UIs). The MENA region is comprised of the following countries: Afghanistan, Algeria, Bahrain, Egypt, Iran, Iraq, Jordan, Kuwait, Lebanon, Libya, Morocco, Oman, Palestine, Qatar, Saudi Arabia, Sudan, the Syrian Arab Republic, Tunisia, Turkey, the United Arab Emirates and Yemen. The population of the MENA region was estimated to be 608.7 million in 2019 [10]. Previous publications provide a detailed description of the methods used to estimate the disease burden for female breast cancer, as well as other diseases and injuries [8, 9]. The results can be accessed online (https://ghdx.healthdata.org/gbd-results-tool). This article was based on a secondary analysis of GBD data and the study was conducted in accordance with the Guidelines for Accurate and Transparent Health Estimates Reporting (Table S1) [11].

### Estimation framework

The International Classification of Diseases (ICD) 10 codes (i.e., C50-C50.9, D05-D05.9, D24-D24.9, D48.6, D49.3) were included in the breast cancer estimates [8]. There were six breast cancer sequalae with different disability weights (DWs) (Table S2) [8]. The data sources used to estimate the non-fatal and fatal burden of female breast cancer included: vital registration, vital registration-sample, verbal autopsy and cancer registries [8].

### Mortality estimation

Mortality-to-incidence ratios (MIR) were used to transform incidence data to mortality estimates, as globally there was less cancer mortality than incidence data available. The ratios were calculated using linear-step mixed effect models in the locations which had both incidence and death data for the same year and these were corrected for sex, age, and Health Care Access and Quality (HAQ). These estimates were smoothed across space and time using Spatio-Temporal Gaussian Processes Regression (ST-GPR) [8]. Mortality estimates were computed by multiplying location-specific MIRs and incidence estimates with each 5-year age group, and sex. The mortality estimates and direct mortality data (i.e., from vital registration and verbal autopsies) were included in the Cause of Death Ensemble Model (CODEm), which was used to identify the best fitting model that could be obtained using all available data and covariates [8].

### Incidence, prevalence and disability estimation

The final incidence estimates were calculated by dividing the CODEm produced breast cancer mortality estimates by their corresponding MIRs. The prevalence of female breast cancer was estimated using the MIRs in each country to estimate survival. Prevalence was split into five sequalae, but mastectomy was not included (Table S2). Years lived with disability (YLDs) were calculated by multiplying the sequelae-specific prevalence with their corresponding DWs Furthermore, the YLDs due to mastectomy were calculated for breast cancer and added to the YLDs (Table S2). The estimated number of deaths by age were then multiplied by a standard life expectancy at that age, in order to calculate the years of life lost (YLLs). Finally, the YLDs and YLLs were summed to estimate the DALYs. All estimates were reported along with 95% uncertainty intervals (UIs).

The relationship between the burden of female breast cancer, in terms of DALYs, and the SDI for all countries located in the MENA region was also assessed using Smoothing Spline models [12]. SDI is an indicator of socio-economic development and is comprised of the lag-dependent income per capita, average years of education for the population aged 15+, and total fertility rate under 25 years of age. The SDI ranges from 0 (less developed) to 1 (most developed) [8].

### Risk factors

This study also reported the proportion of female breast cancer DALYs that were attributable to the following risk factors: alcohol consumption, high fasting plasma glucose, high body mass index, secondhand smoke, smoking and low physical activity. The definitions of these risk factors and their relative risk for breast cancer have previously been reported [9].

## Results

### Middle East and North Africa region

In 2019, there were 94.7 thousand (95% UI: 82.3, 108.9) incidence cases of female breast cancer in the MENA region, with an age-standardised incidence rate of 37.5 (95% UI: 32.7, 42.9) per 100,000, which had increased by 90.9% (95% UI: 54.6, 122.1) since 1990 (Table 1 and Table S3). Female breast cancer accounted for more than 35.4 thousand (95% UI: 30.7, 40.6) deaths, with an age-standardised death rate of 15.2 (95% UI: 13.3, 17.3) per 100,000, which was 24.0% (95% UI: − 0.8, 45.6) higher than in 1990 (Table 1 and Table S4). Moreover, in 2019 there were 1.2 million (95% UI: 1.1, 1.4) DALYs attributable tofemale breast cancer, with an age-standardised rate of 472.7 (95% UI: 409.0, 544.8) per 100,000 females, which increased by 19.5% (95% UI: − 3.2, 40.5) between 1990 and 2019 (Table 1 and Table S5).

**Table 1:** Incidence cases, deaths and disability-adjusted life years(DALYs) due to female breast cancer in 2019 and percentage change of age-standardised rates during 1990–2019 (Generated from data available from http://ghdx.healthdata.org/gbd-results-tool)

	Incidence (95% UI)			Death (95% UI)			DALY (95% UI)		
	Counts (2019)	ASRs (2019)	PCs in ASRs 1990–2019	Counts (2019)	ASRs (2019)	PCs in ASRs 1990–2019	Counts (2019)	ASRs (2019)	PCs in ASRs 1990–2019
**North Africa and Middle East**	**94,746 (82,334, 108,875)**	**37.5 (32.7, 42.9)**	**90.9 (54.6, 122.1)**	**35,405 (30,676, 40,571)**	**15.2 (13.3, 17.3)**	**24 (−0.8, 45.6)**	**1,222,835 (1,053,073, 1,411,009)**	**472.7 (409, 544.8)**	**19.5 (−3.2, 40.5)**
**Afghanistan**	**1951 (1451, 2561)**	**22.3 (16.8, 29.1)**	**30.7 (−8.5, 79.5)**	**1281 (964, 1673)**	**16.5 (12.5, 21.3)**	**18.8 (−16.2, 61.9)**	**48,258 (36,046, 63,895)**	**506.2 (380.9, 663.4)**	**13.3 (− 21.5, 57.5)**
**Algeria**	**6621 (4954, 8490)**	**34 (25.6, 43.3)**	**53.9 (12.2, 107.9)**	**2407 (1823, 3047)**	**13.9 (10.7, 17.3)**	**4.7 (− 21.8, 37.1)**	**83,225 (62,174, 107,206)**	**419.3 (315.9, 537.8)**	**1.9 (− 26.7, 38.5)**
**Bahrain**	**346 (271, 433)**	**67.5 (54, 83.1)**	**47.5 (12.2, 91)**	**103 (82, 127)**	**25.2 (20.4, 30.5)**	**−8.1 (− 28.5, 16.6)**	**3592 (2857, 4487)**	**668.3 (533.3, 827.7)**	**− 15.2 (− 36, 10)**
**Egypt**	**10,600 (7356, 14,525)**	**29.3 (20.1, 40)**	**106.5 (39.4, 188.6)**	**4650 (3159, 6344)**	**14.2 (9.6, 19.2)**	**49 (− 2, 106.6)**	**163,089 (112,703, 223,017)**	**436.2 (299.4, 592)**	**40.8 (− 4.4, 98.3)**
**Iran (Islamic Republic of)**	**14,743 (13,248, 16,469)**	**34 (30.7, 37.9)**	**81.2 (34.6, 130.5)**	**4704 (4306, 5192)**	**11.9 (10.8, 13.1)**	**14.9 (− 15.1, 47.7)**	**161,486 (147,227, 177,500)**	**368.7 (336.7, 404.3)**	**15.2 (− 11.7, 42.5)**
**Iraq**	**7819 (5733, 10,484)**	**52 (38.9, 68.9)**	**79.4 (13.5, 170.3)**	**2970 (2217, 3930)**	**21.6 (16.4, 28.1)**	**24.7 (− 19.5, 88.6)**	**109,032 (79,889, 147,823)**	**714.9 (529.9, 957.2)**	**22.5 (− 21.6, 85)**
**Jordan**	**2053 (1576, 2636)**	**52.9 (41, 67.4)**	**47.6 (7, 105.9)**	**674 (526, 858)**	**19.9 (15.6, 24.9)**	**− 5.1 (− 31, 33.8)**	**23,176 (17,862, 29,882)**	**583.6 (452.8, 744.7)**	**−11.6 (− 35.5, 24.1)**
**Kuwait**	**716 (563, 923)**	**42.8 (34.4, 54.7)**	**3.4 (− 18.3, 36.7)**	**168 (135, 215)**	**13 (10.6, 16.6)**	**− 26.7 (− 41.3, − 4.2)**	**6066 (4831, 7893)**	**358.8 (290.3, 461.8)**	**−31.8 (− 45.8, − 10.3)**
**Lebanon**	**3519 (2655, 4617)**	**122.5 (92.1, 160.7)**	**152.9 (76, 255.6)**	**1012 (777, 1320)**	**35.5 (27.2, 46.4)**	**36.4 (− 5.3, 90.1)**	**30,575 (23,260, 40,346)**	**1067 (808.6, 1407.3)**	**31 (− 9.3, 85.6)**
**Libya**	**1347 (944, 1886)**	**41.4 (29.3, 56.8)**	**96.9 (20.5, 211.2)**	**507 (364, 693)**	**17.2 (12.5, 23.2)**	**46.7 (−8.3, 126.5)**	**18,190 (12,936, 25,290)**	**550.5 (393.6, 753.1)**	**45.9 (− 8.4, 127.9)**
**Morocco**	**9755 (7043, 13,518)**	**52.5 (38.2, 72)**	**86.3 (26.1, 172.5)**	**4372 (3195, 5942)**	**24.4 (18.1, 32.8)**	**32.6 (− 9.2, 87.7)**	**158,502 (114,796, 219,730)**	**842.5 (612.4, 1157.9)**	**30 (− 11.9, 89.9)**
**Oman**	**427 (346, 511)**	**44.7 (36.8, 52.9)**	**131.5 (45.4, 263.8)**	**124 (102, 147)**	**15.9 (13.3, 18.9)**	**45.2 (− 9.3, 127.4)**	**4235 (3446, 5087)**	**434.8 (359.1, 519)**	**33.4 (−15, 107.8)**
**Palestine**	**840 (688, 1012)**	**57.1 (46.9, 68.8)**	**70.7 (6.7, 159)**	**336 (278, 402)**	**25.5 (21.2, 30.5)**	**32.6 (− 16.2, 100.3)**	**11,138 (9150, 13,244)**	**738.9 (609.5, 879)**	**24.1 (− 22.4, 85.9)**
**Qatar**	**382 (283, 501)**	**103.7 (80.2, 131.2)**	**112 (47.2, 201.3)**	**86 (64, 111)**	**36.9 (28.9, 45.8)**	**30.8 (− 8.8, 87)**	**3325 (2493, 4364)**	**856.4 (662.5, 1074.6)**	**7.4 (− 24.6, 50.5)**
**Saudi Arabia**	**5330 (3833, 7262)**	**43.1 (31.9, 57.1)**	**189.8 (79, 358.9)**	**1410 (1032, 1868)**	**14.3 (10.9, 18.7)**	**34.3 (− 17.3, 102.2)**	**56,033 (40,441, 75,439)**	**446.1 (332.2, 589.5)**	**32.6 (− 18.4, 102.1)**
**Sudan**	**2846 (1827, 4005)**	**24 (16.2, 33)**	**75.8 (11.5, 169)**	**1375 (931, 1914)**	**13.1 (9.4, 17.7)**	**32.4 (− 10.9, 100.6)**	**50,738 (32,468, 73,398)**	**416.4 (282.4, 577.1)**	**27.3 (− 17.9, 95.3)**
**Syrian Arab Republic**	**1873 (1303, 2628)**	**26.9 (18.9, 37.3)**	**96.8 (19.6, 219.8)**	**711 (500, 1004)**	**11.3 (8.1, 15.5)**	**36.4 (− 16.2, 121.7)**	**24,006 (16,758, 34,377)**	**334 (237.3, 472.1)**	**25.4 (− 23.8, 101.2)**
**Tunisia**	**3129 (2241, 4237)**	**46 (33, 62.2)**	**84 (18.8, 169.8)**	**1039 (753, 1386)**	**15.7 (11.5, 20.9)**	**12.6 (− 26, 62.9)**	**33,143 (23,657, 44,761)**	**483.5 (345.2, 650.3)**	**14.5 (− 25.9, 66.1)**
**Turkey**	**17,130 (13,440, 21,566)**	**36.1 (28.3, 45.5)**	**85.9 (30.6, 158.8)**	**5926 (4729, 7337)**	**12.6 (10.1, 15.7)**	**3.1 (− 25.9, 40.7)**	**176,292 (138,548, 222,264)**	**369.8 (291, 464.6)**	**− 4 (− 32.1, 33.4)**
**United Arab Emirates**	**1220 (872, 1648)**	**57.5 (43.3, 73.7)**	**41 (− 6.9, 112.1)**	**430 (313, 571)**	**26.2 (20, 33.6)**	**2.8 (− 32.7, 52.5)**	**17,621 (12,543, 23,549)**	**791 (594, 1020.9)**	**6.3 (− 29.9, 59.9)**
**Yemen**	**2001 (1418, 2805)**	**22.7 (16.6, 31.3)**	**79.5 (11.6, 221.9)**	**1085 (790, 1506)**	**13.4 (10, 18.4)**	**46.9 (− 6.7, 159.1)**	**39,870 (28,352, 56,502)**	**434.1 (314.8, 605.8)**	**45.9 (− 9.5, 161.1)**

Abbreviations: *DALY* disability-adjusted life year, *ASRs* age-standardised rates, *PCs* percent changes, *UI* uncertainty interval

### National level

The age-standardised incidence rate of female breast cancer varied substantially between countries in the MENA region. Lebanon [122.5 (95% UI: 92.1, 160.7)], Qatar [103.7 (95% UI: 80.2, 131.2)] and Bahrain [67.5 (95% UI: 54.0, 83.1)] had the highest age-standardised incidence rates per 100,000 females. In contrast, Afghanistan [95% UI: 22.3 (16.8, 29.1)], Yemen [95% UI: 22.7 (16.6, 31.3)] and Sudan [95% UI: 24 (16.2, 33)] had the lowest rates (Table S3). Qatar [36.9 (95% UI: 28.9, 45.8)], Lebanon [35.5 (95% UI: 27.2, 46.4)] and the United Arab Emirates (UAE) [26.2 (95% UI: 20.0, 33.6)] had the three highest age-standardised death rates in 2019, whereas Syria [11.3 (95% UI: 8.1, 15.5)], Iran [11.9 (95% UI: 10.8, 13.1)] and Turkey [12.6 (95% UI: 10.1, 15.7)] had the lowest (Table S4). The highest age-standardised DALY rates in 2019 were observed in Lebanon [1067.0 (95% UI: 808.6, 1407.3)], Qatar [856.4 (95% UI: 662.5, 1074.6)] and Morocco [842.5 (95% UI: 612.4, 1157.9)]. In contrast, Syria [334.0 (95% UI: 237.3, 472.1)], Kuwait [358.8 (95% UI: 290.3, 461.8)] and Iran [368.7 (95% UI: 336.7, 404.3)] had the lowest age-standardised DALY rates (Table S5).

There were substantial differences in the percentage change in the age-standardised incidence rates between 1990 to 2019, with Saudi Arabia [189.8% (95% UI: 79.0, 358.9)], Lebanon [152.9% (95% UI: 76.0, 255.6)] and Oman [131.5% (95% UI: 45.4, 263.8)] having the highest increases, while Kuwait [3.4% (95% UI: − 18.3, 36.7)], Afghanistan [30.7% (95% UI: − 8.5, 79.5)] and the UAE [41.0% (95% UI: − 6.9, 112.1)] had the lowest (Table S3). For the percentage change in the age-standardised death rate, Egypt [49.0% (95% UI: − 2.0, 106.6)], Yemen [46.9% (95% UI: − 6.7, 159.1)] and Libya [46.7% (95% UI: − 8.3, 126.5)] had the largest increases over this period, while Kuwait [− 26.7% (95% UI: − 41.3, − 4.2)], Bahrain [− 8.1% (95% UI: − 28.5, 16.6)] and Jordon [− 5.1% (95% UI: − 31.0, 33.8)] were the only countries with decreased death rates (Table S4). For the percentage change in the age-standardised DALY rates from 1990 to 2019, the largest increases were seen in Yemen [45.9% (95% UI: − 9.5, 161.1)], Libya [45.9% (95% UI: − 8.4, 127.9)] and Egypt [40.8% (95% UI: − 4.4, 98.3)]. In contrast, Kuwait [− 31.8% (95% UI: − 45.8, − 10.3)], Bahrain [− 15.2% (95% UI: − 36.0, 10.0)] and Jordan [− 11.6% (95% UI: − 35.5, 24.1)] had the largest decreases in the DALY rates (Table S5). The trends in the age-standardised incidence, death and DALY rates of female breast cancer in the MENA countries are presented in Figs. S1, S2, and S3.

### Age and sex patterns

**Fig. 1:**
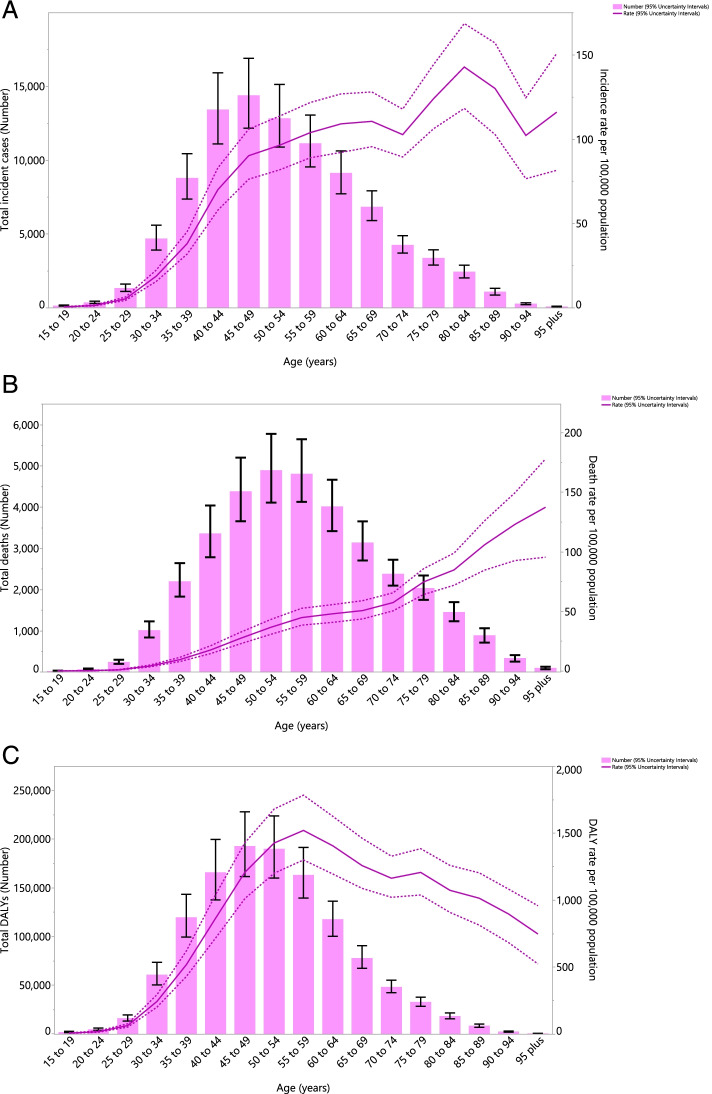
Numbers of incidence cases and incidence rate (**A**), number of deaths and death rate (**B**) and the number of DALYs and DALY rate (**C**) for female breast cancer per 100,000 in the Middle East and North Africa region, by age in 2019 (Generated from data available from http://ghdx.healthdata.org/gbd-results-tool)

In 2019, the number of incidence cases increased with population aging, reaching its peak in the 45–49 age group and then decreased with advancing age. The incidence rate per 100,000 females increased consistently from the early ages up to the older ages, except in the 70–74 age group, which showed a decrease, before increasing again to its peak in the 80–84 age group, before decreasing again (Fig. 1A). Furthermore, the total number of deaths attributable to female breast cancer increased by age up to the 50–54 age group, followed by a decrease for the rest of the age groups. However, the death rate increased constantly with age and peaked in the oldest age group (Fig. 1B). The total number of DALYs associated with female breast cancer increased with aging, peaking in the 45–49 age group and then decreasing with increasing age. The DALY rate also increased steeply with age and reached its highest in the 55–59 age group, followed by a steady decline (Fig. 1C).

**Fig. 2:**
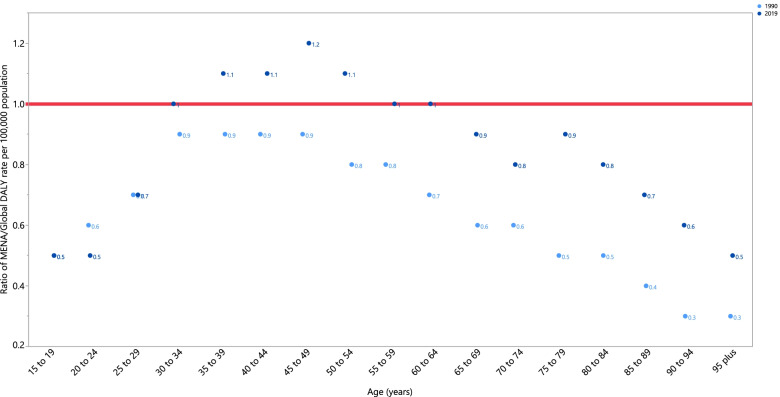
Ratio of the Middle East and North Africa region to the global female breast cancer DALY rate according to age group, 1990–2019. DALY = disability-adjusted-life-year. (Generated from data available from http://ghdx.healthdata.org/gbd-results-tool)

In 2019, females younger than 30 and older than 65 had DALY rates that were lower than the global average (ratio of MENA/global DALY rate < 1). Females aged 30–34 and 55–64 had DALY rates that were similar to the global rate (ratio of MENA/global DALY rate = 1), while those aged 35–54 had higher DALY rates than the global average (ratio of MENA/global DALY rate > 1). It is worth noting that in 2019 the 45–49 age group had the highest ratio (1.2), while the 15–24 and 95^+^ age groups had the lowest ratios (0.5). Compared to 1990, in 2019 females age 30 and older had higher DALY rates in all age groups. Furthermore, while the DALY rates did not change between 1990 and 2019, for females aged 15–19 and 25–29, they decreased for 20–24 year olds (Fig. 2).

### Association with socio-demographic index (SDI)

**Fig. 3:**
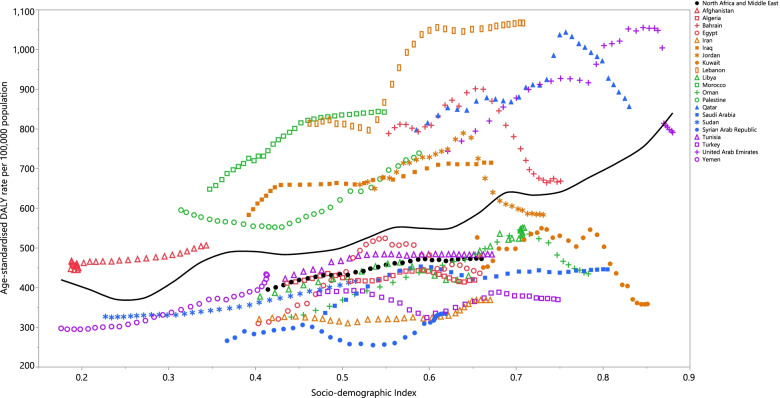
Age-standardised DALY rates of female breast cancer for 21 countries and territories, by SDI in 2019; Expected values based on the Socio-demographic Index and disease rates in all locations are shown as the black line. Each point shows the observed age-standardised DALY rate for each country in 2019. DALY = disability-adjusted-life-years. SDI = Socio-demographic Index (Generated from data available from http://ghdx.healthdata.org/gbd-results-tool)

At the regional level, from 1990 to 2019 there was a positive association between SDI and the DALY rate of female breast cancer. Afghanistan, Palestine, Morocco, Lebanon, Iraq, Bahrain and Qatar had observed rates that were higher than expected, from 1990 to 2019, while Yemen, Sudan, Syria, Iran, Turkey, Saudi Arabia, Algeria, Oman, Libya, Tunisia, Egypt and Kuwait had lower than expected rates, based upon their SDI. Moreover, Jordan and the UAE reached a lower than expected rate during this time period (Fig. 3).

### Risk factors

**Fig. 4:**
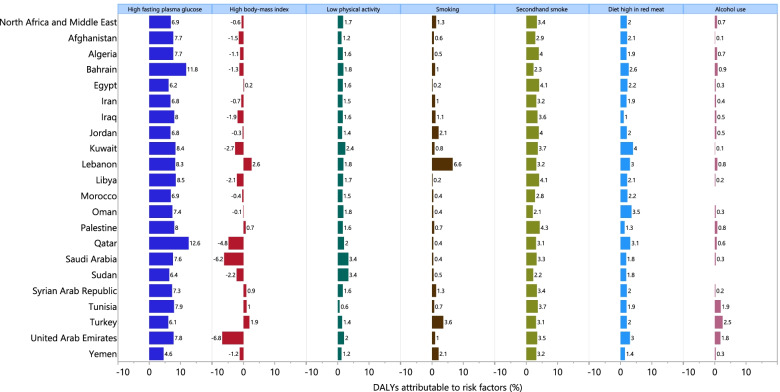
Percentage of DALYs due to female breast cancer attributable to risk factors for the Middle East and North Africa countries in 2019. DALY = disability-adjusted-life-year (Generated from data available from http://ghdx.healthdata.org/gbd-results-tool)

Although there were inter-country differences in the percentage of DALYs attributable to female breast cancer in MENA, the three largest contributors were high fasting plasma glucose (6.9%), second-hand smoke (3.4%) and a diet high in red meat (2.0%). Although high fasting plasma glucose had the largest attributable burden in almost all of the MENA countries, in Lebanon smoking had the largest attributable burden. Conversely, high body mass index was the only risk factor that had a protective effect for female breast cancer, decreasing the attributable DALYs by 0.6%. (Fig. 4). However, a heterogeneous pattern was observed between age and the attributable DALYs caused by high body mass index. More specifically, while high body mass index was associated with higher DALYs up to the 50–54 age group, it resulted in a lower burden for those in older age groups. Additionally, the highest percentage of attributable DALYs due to high fasting plasma glucose (13.6%), second-hand smoke (3.7%) and a diet high in red meat (2.1%) were seen among the 75–79, 45–54, and 25–44 age groups, respectively (Fig. S4).

## Discussion

The current study investigated the burden of female breast cancer in MENA and its attributable risk factors by age and socioeconomic development, and found a substantial increase in the burden of this disease over the last three decades. Compared with the global values in 1990 and 2019, the MENA region had lower age-standardised DALY rates in almost all age group except those aged between 35 and 55 years old. Furthermore, the age-standardised death rate increased with advancing age, while the DALY rates peaked in the 55–59 age group. There was a positive association between the burden of female breast cancer and SDI, while high-fasting plasma glucose accounted for the largest attributable burden.

An article on the global burden of breast cancer, using data from the GBD 2019 project, found the age-standardised rates for the incidence, death and DALY to be 24.2, 8.6 and 247.6 per 100,000, respectively [2], whereas the rates we found were 37.5, 15.2 and 472.7, respectively. The GBD 2017 study on the burden of breast cancer at the global level revealed similar findings to our results [6]. The discrepancies between the two previously mentioned studies and our findings could be due to the different methodologies used for estimating the attributable burden, that we only included females and the fact that we only covered the MENA region. In addition, comparing the breast cancer DALYs found in MENA to the global DALYs showed that MENA had lower incidence, death and DALY rates than those found at the global level [2, 6]. The estimated annual percent of change for the incidence, death and DALYs attributable to breast cancer at the global level were 0.3, − 0.6% and − 0.5%, respectively. In addition, the relative changes in the number of incident cases, deaths and DALYs were 128.3%, 84.0% and 76.6% higher, respectively. Furthermore, previous research reported that in MENA the death rate from breast cancer increased from 6.9 in 1990 to 9.7 in 2015 [13].

At the national level, Lebanon had the highest age-standardised incidence and DALY rates, while Qatar had the largest age-standardised death rate. The results of a study using data from the National Cancer Registry of Lebanon, from 2005 to 2015, showed that breast cancer was the leading form of cancer in Lebanon, accounting for 37% of cancers among females and having a mean age-standardised rate of 91.7 per 100,000 over this period [14]. The same study also compared the incidence of breast cancer with other countries in this region and found that Lebanon had the highest incidence rates of female breast cancer, followed by Malta, Kuwait and Qatar (79.0, 56.1 and 53.8 per 100,000 population, respectively) [14]. In addition, an article about breast cancer from 2004 reported an age-standardised incidence rate of 71.0 per 100,000 in Lebanon, which accounted for 38.2% of all cancer cases among female, which was lower than in developed countries [15]. According to the GLOBOCAN 2012 findings, Lebanon had the highest incidence rates of all types of cancers in the Eastern Mediterranean Region (204.0 and 193.0 per 100,000 individuals in males and females, respectively) [16]. The differences between the countries, in terms of the incidence and burden of breast cancer, could be due to the different levels of exposure or the prevalence of risk factors in each country, implementation of screening programs, accessibility to the screening programs in different areas of a country, willingness of people to participate in these programs or improvements in the cancer registries [14, 17]. Moreover, Lebanon has developed breast cancer screening guidelines for females over 40 years of age, which recommends annual mammography for those without a family history of breast cancer and annual screening 10 years before the age at which cancer was first diagnosed in those with a family history of breast cancer [18]. Furthermore, since 2002 the Lebanese Ministry of Public Health has held annual screening and awareness campaigns for females between October and December [18]. The effects of conflict and turbulence in the region must also be taken into consideration. There have been a number of conflicts in the region, including in Lebanon, Syria, Iraq and Yemen, which have imposed cultural, logistical and financial barriers for cancer care and research [19]. Moreover, the consequences of these conflicts, such as acute injuries, the displacement of sections of the population and the destruction of infrastructure could also affect the provision of medical care and preventive measures, which may also lead to variations in the morbidity and mortality of cancers in MENA [20, 21].

Our findings showed the highest age-standardised incidence, death and DALY rates were in the 80–84, 95+ and 55–59 age groups, respectively. Similarly, an article about Lebanese women showed that the average age-specific incidence rate of breast cancer peaked in the 50–54 age group [14]. Moreover, a global level study showed that most incident cases and deaths from breast cancer occurred between 50 and 70 years of age [2]. Therefore, adult females older than 50 years of age have a higher risk of breast cancer development and morbidity, meaning that screening programs should particularly target this population.

We found a positive association between the age-standardised DALY rate and SDI over the last three decades in MENA. Furthermore, in a study which reported the incidence and deaths for 29 types of cancers at the global, regional and national levels using the GBD 2019 data, they found a positive correlation between breast cancer morbidity and socioeconomic development (i.e., SDI), but a negative relationship between SDI and the fatality rate [2]. Moreover, a similar relationship was found between SDI and the age-standardised incidence and death rates of breast cancer in GBD 2016 [22]. In addition, there was a significant positive correlation between SDI and the age-standardised incidence rate of breast cancer, between 1990 and 2017, in the 21 GBD regions [2]. Although urbanisation and increased exposure to risk factors in more developed countries could lead to this increase, developed countries also have better cancer registers, which might increase the recorded incidence of breast cancer and therefore lead to finding this association. The age-standardised death and DALY rates decreased between 1990 and 2017 in high and high-middle SDI countries, despite an increase in other SDI quintiles [7]. The development of new cancer treatments, like immunotherapy and personalised medicine, could increase the quality of life and decrease mortality and morbidity in high SDI countries.

Among the risk factors of breast cancer in females, high fasting plasma glucose (6.9%) and first−/second-hand smoking (4.7%) were the largest contributors in MENA. At the global level, in 2019 the largest contributor to breast cancer deaths was high fasting plasma glucose, whereas low physical activity and having a diet high in red meat were the lowest contributors [2]. Moreover, over last three decades there were global increases in the deaths attributable to high fasting plasma glucose and high body mass index [2]. In 2017, alcohol consumption (9.4%), high fasting plasma glucose (6.1%) and high body mass index (4.6%) had the highest attributable DALYs due to breast cancer [6]. The population-attributable fraction of high fasting plasma glucose increased from 2.4% of the DALYs from all causes in 1990 to 4.9% in 2013 [23]. Qatar (12.6%) and Bahrain (11.8%), which had the two highest DALYs attributable to high fasting plasma glucose, also had the highest age-standardised prevalence of type 2 diabetes mellitus in MENA (16,312.4 and 14,234.9 per 100,000 in Qatar and Bahrain, respectively) [24]. The non-adherence to medication and suboptimal control of blood glucose levels, which could lead to disease complications, are major problems in MENA. Therefore, it is important to highlight the need for improving disease management and diabetes prevention at the different levels of prevention, especially at the primary prevention level [25]. Our results also showed that in 2019 there was a higher proportion of DALYs attributable to second-hand smoking than first-hand smoking (3.4% vs. 1.3%). This may be due to the higher age-standardised prevalence rate of smoking in males than among females (32.4 vs. 5.6 per 100,000 population) in MENA, and more passive exposure among women [26]. Recommendations for smoking cessation in MENA include higher taxes on tobacco products, inserting warning labels, improving consumer information, as well as behavioral and pharmacological-based interventions [27]. The breast cancer attributable DALYs to high fasting plasma glucose, high body mass index and low physical activity were higher in those above 50 years of age, while second-hand smoking, a diet high in red meat and alcohol consumption were larger contributors among younger females in MENA. This information could be used by policymakers and health authorities at the regional and national levels for the focusing of prevention programs and healthcare planning in this region.

## Strength and limitations of this study

Although previous studies have evaluated the burden of breast cancer at the global level [2, 6], this is one of the first studies to report the burden of female breast cancer and its attributable risk factors in the MENA region, and the first to do this using data from the GBD 2019 project. Nevertheless, the present study has several limitations which should be taken into account when interpreting the findings. Firstly, similar to other GBD papers, the most important limitation is in the data sources used to estimate the burden of disease. Limitations in the cancer registries, data collection and coding, especially in low and middle income countries in the MENA region, could lead to unusable data and data sparsity. The current challenges reducing the accuracy of cancer registries in the region include: poor health infrastructures, the absence of rules mandating cancer registry use, the loss of funding or wars which disrupt the registries, as well as high refugee mobility [28]. It is important to note that the GBD project uses modelling strategies to estimate the burden of diseases and injuries, since the actual data are not likely to be available for less developed countries. Secondly, the GBD project did not report the attributable burden to several other risk factors, including genetic risk factors, early menarche, late menopause and menopausal hormone therapy [29, 30]. Furthermore, the attributable burden was reported for only one type of diet (i.e. red meat), while other dietary patterns and micro- and macro-nutrients, such as carbohydrates, fiber, fruits and vegetables, fatty acids and vitamin D could not be reported in the present study [31]. Thirdly, the incidence, deaths and DALYs attributable to female breast cancer may change due to differences in exposure to the known risk factors and access to screening and therapeutic measures by race/ethnicity and the area of residence [32, 33]. However, the burden of female breast cancer by race and area of residence was not reported in this article, but should be considered in future research. Fourthly, the present study only estimated the burden of breast cancer up to 2019, and therefore, the effects of the COVID-19 pandemic on the burden of breast cancer have not yet been evaluated. This gap needs to be filled in future iterations of the GBD project, because of its importance for the implementation of breast cancer screening programs in future epidemics and pandemics.

## Conclusions

The burden of female breast cancer in MENA has greatly increased in the last three decades. Preventive programs should target the most important risk factors, which are high fasting plasma glucose and smoking, and should be initiated among young women. Additionally, females over 50 years old and those living in countries with higher socioeconomic development should be the priorities for these programs. Further studies are needed to evaluate the effects of health policies on the burden of breast cancer in the individual countries of this region and to estimate its burden for the coming decades, which could be useful information for healthcare authorities.

## Supplementary Information

**Additional file 1: Fig. S1.** Trends in age-standardised incidence rates per 100,000 from 1990 to 2019 in the North Africa and the Middle East region (Generated from data available from http://ghdx.healthdata.org/gbd-results-tool).

**Additional file 2: Fig. S2.** Trends in age-standardised death rates per 100,000 from 1990 to 2019 in the North Africa and the Middle East region (Generated from data available from http://ghdx.healthdata.org/gbd-results-tool).

**Additional file 3: Fig. S3.** Trends in age-standardised DALY rates per 100,000 from 1990 to 2019 in the North Africa and the Middle East region. DALY = disability-adjusted-life-year (Generated from data available from http://ghdx.healthdata.org/gbd-results-tool).

**Additional file 4: Fig. S4.** Percentage of DALYs due to female breast cancer attributable to risk factors for the Middle East and North Africa countries, by age, in 2019. DALY = disability-adjusted-life-year (Generated from data available from http://ghdx.healthdata.org/gbd-results-tool).

**Additional file 5: Table S1.** The completed checklist of the Guidelines for Accurate and Transparent Health Estimates Reporting.

**Additional file 6: Table S2.** Sequelae for female breast cancer and their corresponding disability weights in the GBD 2019 Study.

**Additional file 7: Table S3.** Incidence of female breast cancer in 1990 and 2019 and percentage change in age-standardised rates (ASRs) per 100,000 in the North Africa and the Middle East region (Generated from data available from http://ghdx.healthdata.org/gbd-results-tool).

**Additional file 8: Table S4.** Deaths of female breast cancer in 1990 and 2019 and percentage change in age-standardised rates (ASRs) per 100,000 in the North Africa and the Middle East region (Generated from data available from http://ghdx.healthdata.org/gbd-results-tool).

**Additional file 9: Table S5.** DALYs due to female breast cancer in 1990 and 2019 and percentage change in age-standardised rates (ASRs) per 100,000 in the North Africa and the Middle East region (Generated from data available from http://ghdx.healthdata.org/gbd-results-tool).

## Data Availability

The data used for these analyses are all publicly available at http://ghdx.healthdata.org/gbd-results-tool.
