# Supplementary figures and images for: Burden of female breast cancer in the Middle East and North Africa region, 1990–2019
Source: Arch Public Health. 2022 Jul 11;80:168. doi: 10.1186/s13690-022-00918-y (PMC9272597; doi:10.1186/s13690-022-00918-y)

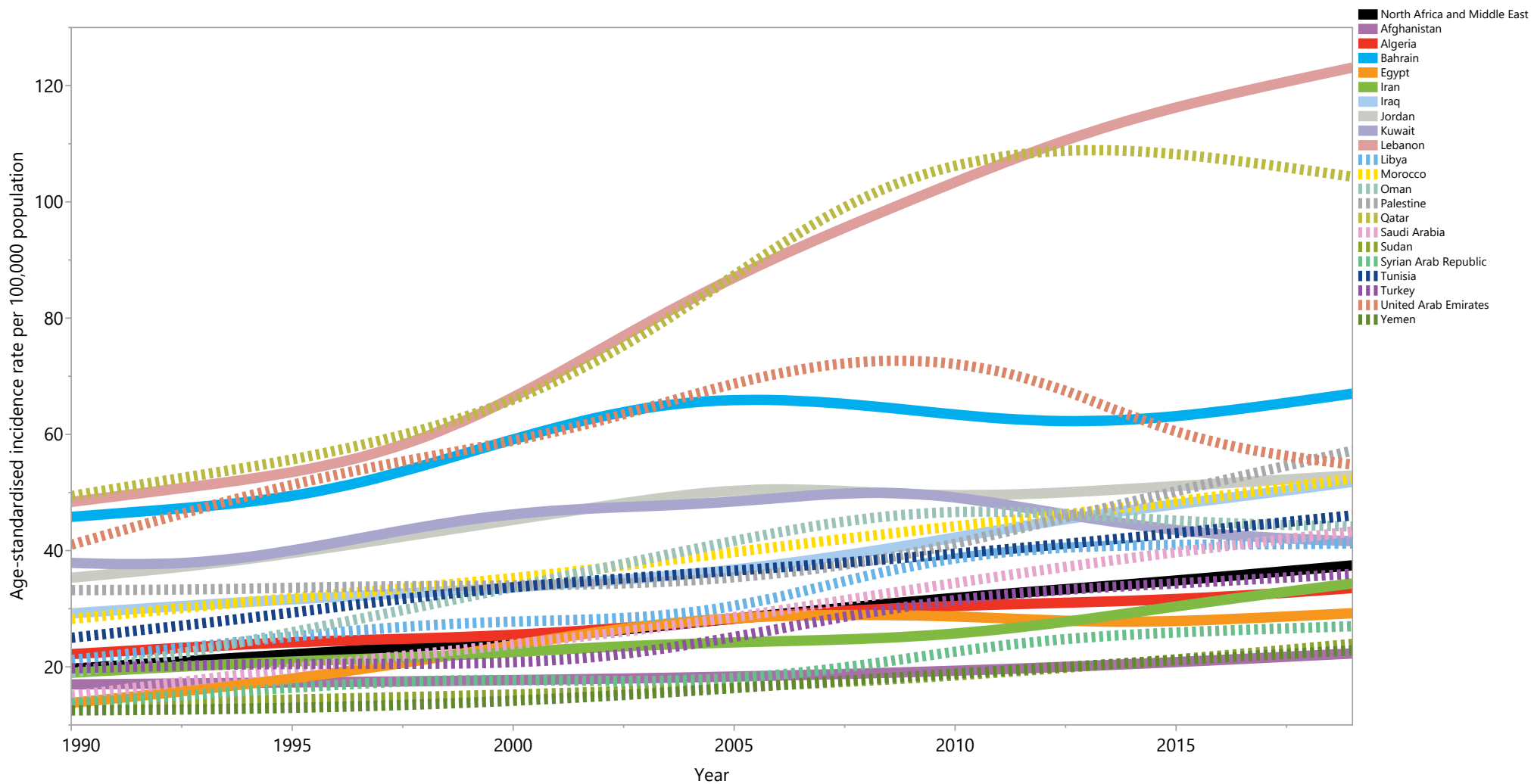

Supplement: Supplementary file 1 — Additional file 1: Fig. S1. Trends in age-standardised incidence rates per 100,000 from 1990 to 2019 in the North Africa and the Middle East region (Generated from data available from http://ghdx.healthdata.org/gbd-results-tool). [file 13690_2022_918_MOESM1_ESM.pdf]

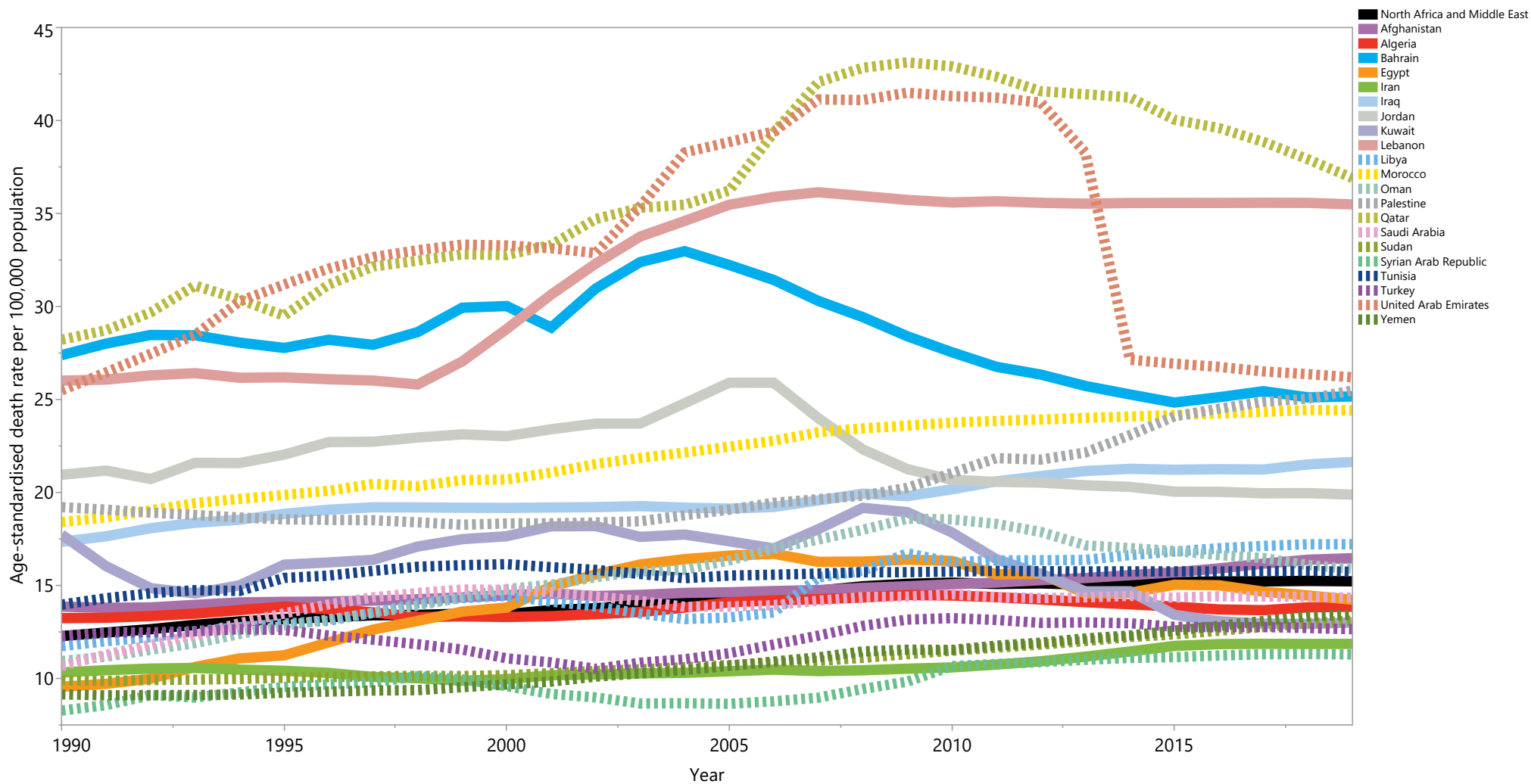

Supplement: Supplementary file 2 — Additional file 2: Fig. S2. Trends in age-standardised death rates per 100,000 from 1990 to 2019 in the North Africa and the Middle East region (Generated from data available from http://ghdx.healthdata.org/gbd-results-tool). [file 13690_2022_918_MOESM2_ESM.pdf]

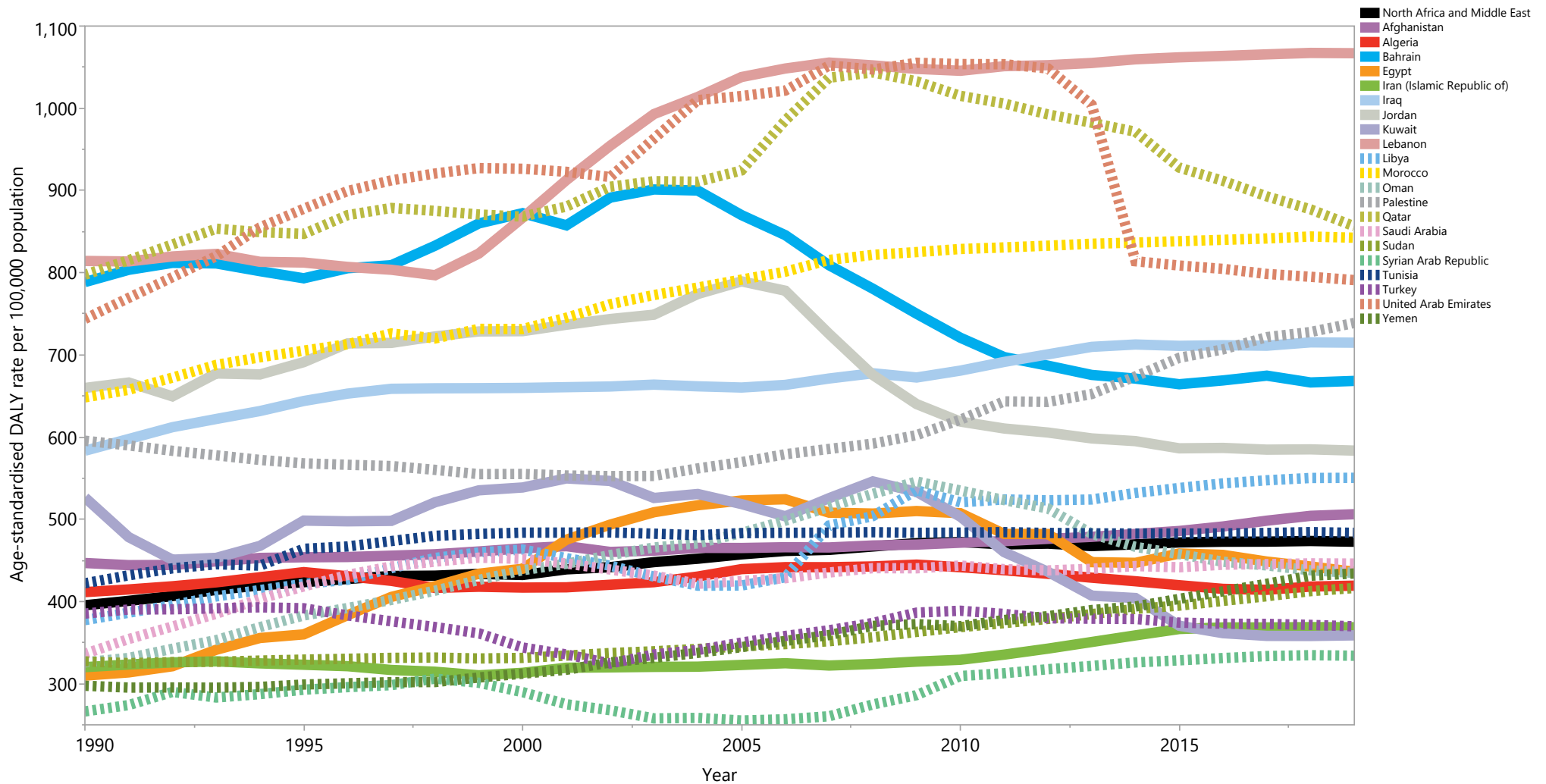

Supplement: Supplementary file 3 — Additional file 3: Fig. S3. Trends in age-standardised DALY rates per 100,000 from 1990 to 2019 in the North Africa and the Middle East region. DALY = disability-adjusted-life-year (Generated from data available from http://ghdx.healthdata.org/gbd-results-tool). [file 13690_2022_918_MOESM3_ESM.pdf]

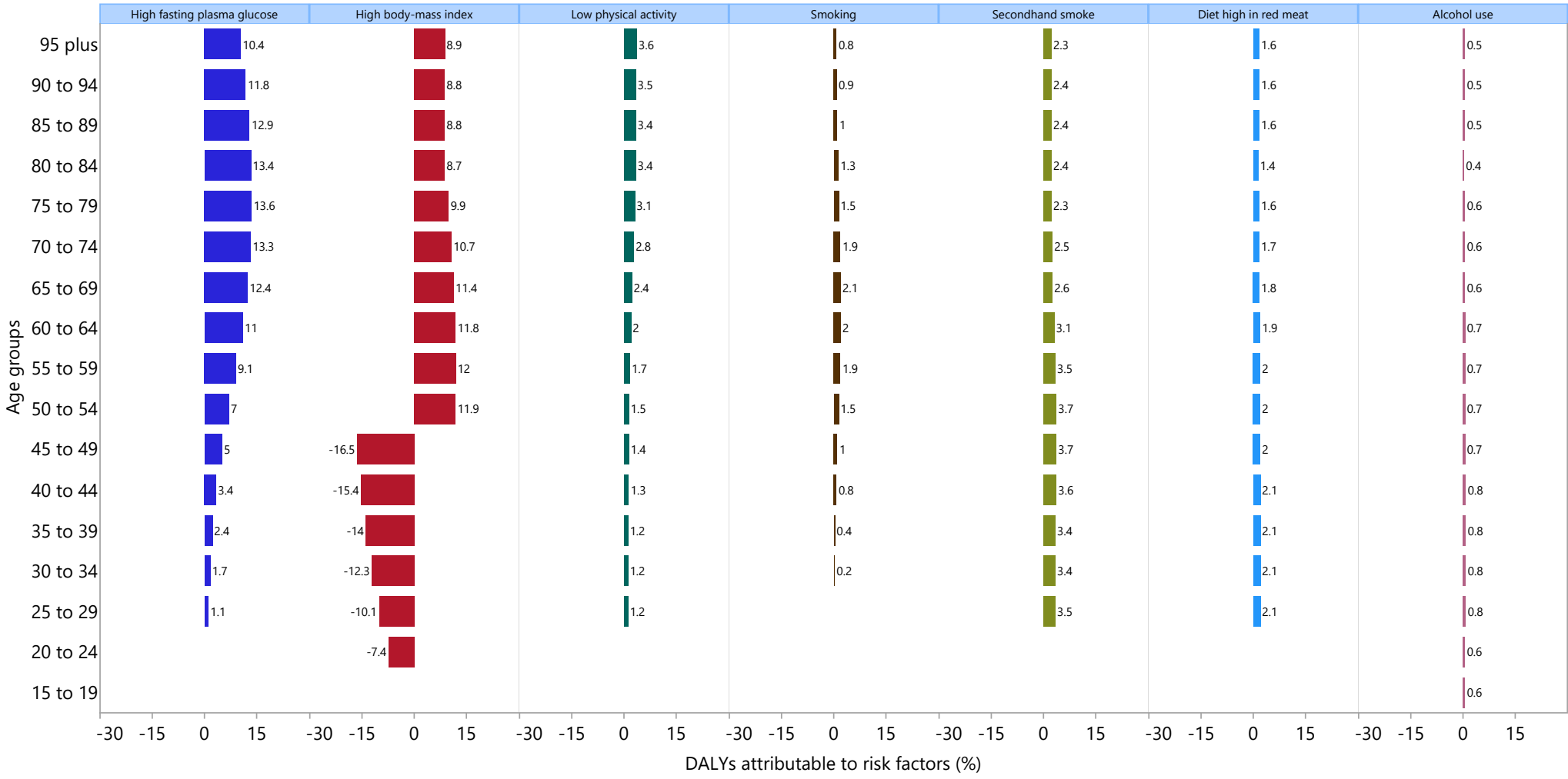

Supplement: Supplementary file 4 — Additional file 4: Fig. S4. Percentage of DALYs due to female breast cancer attributable to risk factors for the Middle East and North Africa countries, by age, in 2019. DALY = disability-adjusted-life-year (Generated from data available from http://ghdx.healthdata.org/gbd-results-tool). [file 13690_2022_918_MOESM4_ESM.pdf]
