# Supplementary material for: Burden of female breast cancer in the Middle East and North Africa region, 1990–2019
Source: Arch Public Health. 2022 Jul 11;80:168. doi: 10.1186/s13690-022-00918-y (PMC9272597; doi:10.1186/s13690-022-00918-y)
Supplement: Supplementary file 6 — Additional file 6: Table S2. Sequelae for female breast cancer and their corresponding disability weights in the GBD 2019 Study. [file 13690_2022_918_MOESM6_ESM.doc]

| **Table S2: Sequelae for female breast cancer and their corresponding disability weights in the GBD 2019 Study** | | | |
| --- | --- | --- | --- |
| **Sequelae** | **Health state name** | **Lay description** | **DW**  **(95% CI)** |
| Diagnosis and primary therapy phase | Cancer, diagnosis and primary therapy | Has pain, nausea, fatigue, weight loss and high anxiety. | 0.288 (0.193-0.399) |
| Controlled phase of breast cancer, without mastectomy | Generic uncomplicated disease: worry and daily  medication | Has a chronic disease that requires medication every day and causes some worry but minimal interference with daily activities. | 0.049 (0.031-0.072) |
| Controlled phase of breast cancer, with mastectomy | Mastectomy and generic medication | (combined DW) | 0.083  (0.052-0.124) |
| Metastatic phase | Cancer, metastatic | Has severe pain, extreme fatigue, weight loss and high anxiety. | 0.451 (0.307-0.600) |
| Terminal phase | Terminal phase, with medication (for cancers, end-stage  kidney/liver disease) | Has lost a lot of weight and regularly uses strong medication to avoid constant pain. The person has no appetite, feels nauseous, and needs to spend most of the day in bed. | 0.540 (0.377-0.687) |
| Mastectomy from breast cancer, beyond 10 years | Mastectomy | had one of her breasts removed and sometimes has pain or swelling in the arms. | 0.036  (0.02-0.057) |
| **GBD: Global Burden of Disease**  **DW: Disability weight** | | | |
