# Supplementary material for: Burden of female breast cancer in the Middle East and North Africa region, 1990–2019
Source: Arch Public Health. 2022 Jul 11;80:168. doi: 10.1186/s13690-022-00918-y (PMC9272597; doi:10.1186/s13690-022-00918-y)
Supplement: Supplementary file 7 — Additional file 7: Table S3. Incidence of female breast cancer in 1990 and 2019 and percentage change in age-standardised rates (ASRs) per 100,000 in the North Africa and the Middle East region (Generated from data available from http://ghdx.healthdata.org/gbd-results-tool). [file 13690_2022_918_MOESM7_ESM.docx]

| **Table S3: Incidence of female breast cancer in 1990 and 2019 and the percentage change in the age-standardised rates (ASRs) per 100,000 in the Middle East and North Africa region**  **(Generated from data available from http://ghdx.healthdata.org/gbd-results-tool)** | | | | | |
| --- | --- | --- | --- | --- | --- |
|  | **1990** | | **2019** | | **Percentage change in ASRs per 100,000** |
|  | **No (95% UI)** | **ASRs per 100,000 (95% UI)** | **No (95% UI)** | **ASRs per 100,000 (95% UI)** |  |
| **North Africa and Middle East** | **19610 (17751 , 22419)** | **19.6 (17.8 , 22.6)** | **94746 (82334 , 108875)** | **37.5 (32.7 , 42.9)** | **90.9 (54.6 , 122.1)** |
| **Afghanistan** | **644 (497 , 829)** | **17.1 (13.4 , 21.7)** | **1951 (1451 , 2561)** | **22.3 (16.8 , 29.1)** | **30.7 (-8.5 , 79.5)** |
| **Algeria** | **1526 (1186 , 1937)** | **22.1 (17.5 , 27.9)** | **6621 (4954 , 8490)** | **34 (25.6 , 43.3)** | **53.9 (12.2 , 107.9)** |
| **Bahrain** | **47 (39 , 56)** | **45.8 (38.9 , 54)** | **346 (271 , 433)** | **67.5 (54 , 83.1)** | **47.5 (12.2 , 91)** |
| **Egypt** | **2509 (2285 , 2749)** | **14.2 (13 , 15.5)** | **10600 (7356 , 14525)** | **29.3 (20.1 , 40)** | **106.5 (39.4 , 188.6)** |
| **Iran (Islamic Republic of)** | **2835 (2352 , 3575)** | **18.8 (15.3 , 24.1)** | **14743 (13248 , 16469)** | **34 (30.7 , 37.9)** | **81.2 (34.6 , 130.5)** |
| **Iraq** | **1337 (970 , 1824)** | **29 (21 , 40)** | **7819 (5733 , 10484)** | **52 (38.9 , 68.9)** | **79.4 (13.5 , 170.3)** |
| **Jordan** | **301 (234 , 377)** | **35.9 (27.6 , 45.2)** | **2053 (1576 , 2636)** | **52.9 (41 , 67.4)** | **47.6 (7 , 105.9)** |
| **Kuwait** | **145 (131 , 162)** | **41.3 (37.6 , 45.8)** | **716 (563 , 923)** | **42.8 (34.4 , 54.7)** | **3.4 (-18.3 , 36.7)** |
| **Lebanon** | **613 (480 , 767)** | **48.4 (38.3 , 60.4)** | **3519 (2655 , 4617)** | **122.5 (92.1 , 160.7)** | **152.9 (76 , 255.6)** |
| **Libya** | **209 (164 , 278)** | **21 (16.4 , 27.8)** | **1347 (944 , 1886)** | **41.4 (29.3 , 56.8)** | **96.9 (20.5 , 211.2)** |
| **Morocco** | **2348 (1891 , 2838)** | **28.2 (22.6 , 33.9)** | **9755 (7043 , 13518)** | **52.5 (38.2 , 72)** | **86.3 (26.1 , 172.5)** |
| **Oman** | **68 (46 , 99)** | **19.3 (13.2 , 28.4)** | **427 (346 , 511)** | **44.7 (36.8 , 52.9)** | **131.5 (45.4 , 263.8)** |
| **Palestine** | **176 (124 , 252)** | **33.5 (23.4 , 47.8)** | **840 (688 , 1012)** | **57.1 (46.9 , 68.8)** | **70.7 (6.7 , 159)** |
| **Qatar** | **30 (23 , 38)** | **48.9 (37 , 64.6)** | **382 (283 , 501)** | **103.7 (80.2 , 131.2)** | **112 (47.2 , 201.3)** |
| **Saudi Arabia** | **464 (331 , 626)** | **14.9 (10.8 , 20.1)** | **5330 (3833 , 7262)** | **43.1 (31.9 , 57.1)** | **189.8 (79 , 358.9)** |
| **Sudan** | **730 (522 , 1021)** | **13.7 (9.7 , 19.5)** | **2846 (1827 , 4005)** | **24 (16.2 , 33)** | **75.8 (11.5 , 169)** |
| **Syrian Arab Republic** | **422 (304 , 567)** | **13.7 (9.8 , 18.1)** | **1873 (1303 , 2628)** | **26.9 (18.9 , 37.3)** | **96.8 (19.6 , 219.8)** |
| **Tunisia** | **680 (557 , 840)** | **25 (20.4 , 30.9)** | **3129 (2241 , 4237)** | **46 (33 , 62.2)** | **84 (18.8 , 169.8)** |
| **Turkey** | **4047 (3201 , 5151)** | **19.4 (15.4 , 24.6)** | **17130 (13440 , 21566)** | **36.1 (28.3 , 45.5)** | **85.9 (30.6 , 158.8)** |
| **United Arab Emirates** | **86 (63 , 119)** | **40.8 (28.7 , 57.8)** | **1220 (872 , 1648)** | **57.5 (43.3 , 73.7)** | **41 (-6.9 , 112.1)** |
| **Yemen** | **378 (229 , 620)** | **12.6 (7.6 , 21)** | **2001 (1418 , 2805)** | **22.7 (16.6 , 31.3)** | **79.5 (11.6 , 221.9)** |
