# Supplementary material for: Burden of female breast cancer in the Middle East and North Africa region, 1990–2019
Source: Arch Public Health. 2022 Jul 11;80:168. doi: 10.1186/s13690-022-00918-y (PMC9272597; doi:10.1186/s13690-022-00918-y)
Supplement: Supplementary file 8 — Additional file 8: Table S4. Deaths of female breast cancer in 1990 and 2019 and percentage change in age-standardised rates (ASRs) per 100,000 in the North Africa and the Middle East region (Generated from data available from http://ghdx.healthdata.org/gbd-results-tool). [file 13690_2022_918_MOESM8_ESM.docx]

| **Table S4: Deaths from female breast cancer in 1990 and 2019 and the percentage change in the age-standardised rates (ASRs) per 100,000 in the Middle East and North Africa region**  **(Generated from data available from http://ghdx.healthdata.org/gbd-results-tool)** | | | | | |
| --- | --- | --- | --- | --- | --- |
|  | **1990** | | **2019** | | **Percentage change in ASRs per 100,000** |
|  | **No (95% UI)** | **ASRs per 100,000 (95% UI)** | **No (95% UI)** | **ASRs per 100,000 (95% UI)** |  |
| **North Africa and Middle East** | **11524 (10398 , 13312)** | **12.3 (11 , 14.2)** | **35405 (30676 , 40571)** | **15.2 (13.3 , 17.3)** | **24 (-0.8 , 45.6)** |
| **Afghanistan** | **501 (389 , 642)** | **13.9 (11 , 17.5)** | **1281 (964 , 1673)** | **16.5 (12.5 , 21.3)** | **18.8 (-16.2 , 61.9)** |
| **Algeria** | **829 (657 , 1045)** | **13.2 (10.8 , 16.4)** | **2407 (1823 , 3047)** | **13.9 (10.7 , 17.3)** | **4.7 (-21.8 , 37.1)** |
| **Bahrain** | **24 (21 , 29)** | **27.4 (23.5 , 31.8)** | **103 (82 , 127)** | **25.2 (20.4 , 30.5)** | **-8.1 (-28.5 , 16.6)** |
| **Egypt** | **1581 (1450 , 1723)** | **9.6 (8.8 , 10.4)** | **4650 (3159 , 6344)** | **14.2 (9.6 , 19.2)** | **49 (-2 , 106.6)** |
| **Iran (Islamic Republic of)** | **1413 (1160 , 1796)** | **10.3 (8.2 , 13.6)** | **4704 (4306 , 5192)** | **11.9 (10.8 , 13.1)** | **14.9 (-15.1 , 47.7)** |
| **Iraq** | **762 (548 , 1046)** | **17.3 (12.5 , 23.8)** | **2970 (2217 , 3930)** | **21.6 (16.4 , 28.1)** | **24.7 (-19.5 , 88.6)** |
| **Jordan** | **160 (123 , 200)** | **21 (15.9 , 26.7)** | **674 (526 , 858)** | **19.9 (15.6 , 24.9)** | **-5.1 (-31 , 33.8)** |
| **Kuwait** | **51 (47 , 55)** | **17.7 (16.1 , 19.4)** | **168 (135 , 215)** | **13 (10.6 , 16.6)** | **-26.7 (-41.3 , -4.2)** |
| **Lebanon** | **313 (250 , 392)** | **26 (21 , 32.3)** | **1012 (777 , 1320)** | **35.5 (27.2 , 46.4)** | **36.4 (-5.3 , 90.1)** |
| **Libya** | **111 (87 , 148)** | **11.7 (9.1 , 15.6)** | **507 (364 , 693)** | **17.2 (12.5 , 23.2)** | **46.7 (-8.3 , 126.5)** |
| **Morocco** | **1472 (1187 , 1766)** | **18.4 (14.9 , 22)** | **4372 (3195 , 5942)** | **24.4 (18.1 , 32.8)** | **32.6 (-9.2 , 87.7)** |
| **Oman** | **35 (24 , 52)** | **10.9 (7.5 , 16.2)** | **124 (102 , 147)** | **15.9 (13.3 , 18.9)** | **45.2 (-9.3 , 127.4)** |
| **Palestine** | **96 (68 , 137)** | **19.2 (13.6 , 27.6)** | **336 (278 , 402)** | **25.5 (21.2 , 30.5)** | **32.6 (-16.2 , 100.3)** |
| **Qatar** | **14 (11 , 17)** | **28.2 (20.9 , 38)** | **86 (64 , 111)** | **36.9 (28.9 , 45.8)** | **30.8 (-8.8 , 87)** |
| **Saudi Arabia** | **307 (219 , 422)** | **10.7 (7.7 , 14.8)** | **1410 (1032 , 1868)** | **14.3 (10.9 , 18.7)** | **34.3 (-17.3 , 102.2)** |
| **Sudan** | **501 (358 , 706)** | **9.9 (7 , 14.4)** | **1375 (931 , 1914)** | **13.1 (9.4 , 17.7)** | **32.4 (-10.9 , 100.6)** |
| **Syrian Arab Republic** | **236 (169 , 312)** | **8.3 (6 , 11)** | **711 (500 , 1004)** | **11.3 (8.1 , 15.5)** | **36.4 (-16.2 , 121.7)** |
| **Tunisia** | **354 (291 , 436)** | **14 (11.5 , 17.3)** | **1039 (753 , 1386)** | **15.7 (11.5 , 20.9)** | **12.6 (-26 , 62.9)** |
| **Turkey** | **2450 (1949 , 3126)** | **12.3 (9.8 , 15.6)** | **5926 (4729 , 7337)** | **12.6 (10.1 , 15.7)** | **3.1 (-25.9 , 40.7)** |
| **United Arab Emirates** | **45 (32 , 62)** | **25.5 (17.9 , 36.3)** | **430 (313 , 571)** | **26.2 (20 , 33.6)** | **2.8 (-32.7 , 52.5)** |
| **Yemen** | **260 (157 , 428)** | **9.1 (5.4 , 15.2)** | **1085 (790 , 1506)** | **13.4 (10 , 18.4)** | **46.9 (-6.7 , 159.1)** |
