# Supplementary material for: Burden of female breast cancer in the Middle East and North Africa region, 1990–2019
Source: Arch Public Health. 2022 Jul 11;80:168. doi: 10.1186/s13690-022-00918-y (PMC9272597; doi:10.1186/s13690-022-00918-y)
Supplement: Supplementary file 9 — Additional file 9: Table S5. DALYs due to female breast cancer in 1990 and 2019 and percentage change in age-standardised rates (ASRs) per 100,000 in the North Africa and the Middle East region (Generated from data available from http://ghdx.healthdata.org/gbd-results-tool). [file 13690_2022_918_MOESM9_ESM.docx]

| **Table S5: DALYs due to female breast cancer in 1990 and 2019 and the percentage change in the age-standardised rates (ASRs) per 100,000 in the Middle East and North Africa region**  **(Generated from data available from http://ghdx.healthdata.org/gbd-results-tool)** | | | | | |
| --- | --- | --- | --- | --- | --- |
|  | **1990** | | **2019** | | **Percentage change in ASRs per 100,000** |
|  | **No (95% UI)** | **ASRs per 100,000 (95% UI)** | **No (95% UI)** | **ASRs per 100,000 (95% UI)** |  |
| **North Africa and Middle East** | **408086 (370190 , 472046)** | **395.4 (357.6 , 458.5)** | **1222835 (1053073 , 1411009)** | **472.7 (409 , 544.8)** | **19.5 (-3.2 , 40.5)** |
| **Afghanistan** | **17555 (13344 , 22678)** | **446.8 (342.1 , 573.5)** | **48258 (36046 , 63895)** | **506.2 (380.9 , 663.4)** | **13.3 (-21.5 , 57.5)** |
| **Algeria** | **29725 (23246 , 37929)** | **411.4 (324.4 , 522.1)** | **83225 (62174 , 107206)** | **419.3 (315.9 , 537.8)** | **1.9 (-26.7 , 38.5)** |
| **Bahrain** | **859 (721 , 1013)** | **788.6 (671 , 925.1)** | **3592 (2857 , 4487)** | **668.3 (533.3 , 827.7)** | **-15.2 (-36 , 10)** |
| **Egypt** | **57072 (51856 , 62539)** | **309.9 (283.4 , 338.1)** | **163089 (112703 , 223017)** | **436.2 (299.4 , 592)** | **40.8 (-4.4 , 98.3)** |
| **Iran (Islamic Republic of)** | **49919 (42208 , 62170)** | **320.2 (265.4 , 405.4)** | **161486 (147227 , 177500)** | **368.7 (336.7 , 404.3)** | **15.2 (-11.7 , 42.5)** |
| **Iraq** | **27485 (19934 , 37671)** | **583.4 (421.5 , 798.3)** | **109032 (79889 , 147823)** | **714.9 (529.9 , 957.2)** | **22.5 (-21.6 , 85)** |
| **Jordan** | **5772 (4486 , 7096)** | **659.8 (507.8 , 815.4)** | **23176 (17862 , 29882)** | **583.6 (452.8 , 744.7)** | **-11.6 (-35.5 , 24.1)** |
| **Kuwait** | **1883 (1719 , 2086)** | **526.5 (481.8 , 578.8)** | **6066 (4831 , 7893)** | **358.8 (290.3 , 461.8)** | **-31.8 (-45.8 , -10.3)** |
| **Lebanon** | **10513 (8225 , 13210)** | **814.3 (642.1 , 1023.2)** | **30575 (23260 , 40346)** | **1067 (808.6 , 1407.3)** | **31 (-9.3 , 85.6)** |
| **Libya** | **3830 (3004 , 5070)** | **377.4 (295.1 , 500.4)** | **18190 (12936 , 25290)** | **550.5 (393.6 , 753.1)** | **45.9 (-8.4 , 127.9)** |
| **Morocco** | **55293 (44320 , 67421)** | **648.3 (519.8 , 783.8)** | **158502 (114796 , 219730)** | **842.5 (612.4 , 1157.9)** | **30 (-11.9 , 89.9)** |
| **Oman** | **1180 (795 , 1718)** | **326 (221.6 , 479.8)** | **4235 (3446 , 5087)** | **434.8 (359.1 , 519)** | **33.4 (-15 , 107.8)** |
| **Palestine** | **3203 (2251 , 4558)** | **595.6 (417.5 , 845.3)** | **11138 (9150 , 13244)** | **738.9 (609.5 , 879)** | **24.1 (-22.4 , 85.9)** |
| **Qatar** | **522 (405 , 667)** | **797.4 (605.2 , 1045.6)** | **3325 (2493 , 4364)** | **856.4 (662.5 , 1074.6)** | **7.4 (-24.6 , 50.5)** |
| **Saudi Arabia** | **11149 (7894 , 15281)** | **336.4 (241.5 , 461.4)** | **56033 (40441 , 75439)** | **446.1 (332.2 , 589.5)** | **32.6 (-18.4 , 102.1)** |
| **Sudan** | **18190 (13030 , 25216)** | **327.1 (234.7 , 460)** | **50738 (32468 , 73398)** | **416.4 (282.4 , 577.1)** | **27.3 (-17.9 , 95.3)** |
| **Syrian Arab Republic** | **8539 (6149 , 11423)** | **266.3 (191.5 , 353.6)** | **24006 (16758 , 34377)** | **334 (237.3 , 472.1)** | **25.4 (-23.8 , 101.2)** |
| **Tunisia** | **11818 (9707 , 14518)** | **422.1 (347.5 , 518.3)** | **33143 (23657 , 44761)** | **483.5 (345.2 , 650.3)** | **14.5 (-25.9 , 66.1)** |
| **Turkey** | **82319 (65124 , 105081)** | **385.3 (306 , 488.2)** | **176292 (138548 , 222264)** | **369.8 (291 , 464.6)** | **-4 (-32.1 , 33.4)** |
| **United Arab Emirates** | **1699 (1239 , 2336)** | **743.8 (526.3 , 1041.7)** | **17621 (12543 , 23549)** | **791 (594 , 1020.9)** | **6.3 (-29.9 , 59.9)** |
| **Yemen** | **9285 (5633 , 15062)** | **297.5 (181.5 , 490.7)** | **39870 (28352 , 56502)** | **434.1 (314.8 , 605.8)** | **45.9 (-9.5 , 161.1)** |
